# Supplementary material for: Polyketide synthase gene clusters in the ericoid mycorrhizal fungus Oidiodendron maius: genome-wide prediction, expression profiling, and functional characterization of knockout mutants
Source: Front Plant Sci. 2026 Sep 16;17:1924430. doi: 10.3389/fpls.2026.1924430 (PMC13623903; doi:10.3389/fpls.2026.1924430)
Supplement: Supplementary file 1 [file SupplementaryFile1.zip › Supplementary Materials/Supplementary Folder 1.zip!/Oidma1_AssemblyScaffolds_Repeatmasked/knownclusterblast/cluster59/jgi.p_Oidma1_139495_mibig_hits.html]

| MIBiG Protein | Description | MIBiG Cluster | MiBiG Product | % ID | % Coverage | BLAST Score | E-value |
| --- | --- | --- | --- | --- | --- | --- | --- |
| AAY93391.1 | phenylacetaldehyde\_dehydrogenase\_PeaE | BGC0000413 | Pyoverdine\_biosynthetic\_gene\_cluster | 35 | 97.3154362416 | 109 | 4.3e-24 |
| CAE45692.1 | hypothetical\_protein | BGC0000031 | Borrelidin\_biosynthetic\_gene\_cluster | 39 | 91.2751677852 | 107 | 1.3e-23 |
| AFY23020.1 | betaine\_aldehyde\_dehydrogenase | BGC0000274 | Tetarimycin\_biosynthetic\_gene\_cluster | 35 | 97.3154362416 | 100 | 2e-21 |
| BAK64640.1 | putative\_aldehyde\_dehydrogenase | BGC0000135 | Reveromycin\_biosynthetic\_gene\_cluster | 33 | 101.342281879 | 97 | 1.3e-20 |
| AAS47555.1 | putative\_aldehyde\_dehydrogenase | BGC0001108 | Pederin\_biosynthetic\_gene\_cluster | 35 | 87.2483221477 | 92 | 5.5e-19 |
| AAW03334.1 | hypothetical\_protein | BGC0000982 | Cystothiazole\_A\_biosynthetic\_gene\_cluster | 31 | 91.9463087248 | 89 | 3.6e-18 |
| BAP16694.1 | aldehyde\_dehydrogenase | BGC0000376 | JBIR-34,\_JBIR-35\_biosynthetic\_gene\_cluster | 33 | 97.3154362416 | 87 | 1e-17 |
